# Relative Roles of Soil Moisture, Nutrient Supply, Depth, and Mechanical Impedance in Determining Composition and Structure of Wisconsin Prairies

**DOI:** 10.1371/journal.pone.0137963

**Published:** 2015-09-14

**Authors:** Robert W. Wernerehl, Thomas J. Givnish

**Affiliations:** 1 Massachusetts State Botanist, Natural Heritage and Endangered Species Program, Westborough, Massachusetts, United States of America; 2 Department of Botany, University of Wisconsin-Madison, Madison, Wisconsin, United States of America; Chinese Academy of Forestry, CHINA

## Abstract

Ecologists have long classified Midwestern prairies based on compositional variation assumed to reflect local gradients in moisture availability. The best known classification is based on Curtis’ continuum index (CI), calculated using the presence of indicator species thought centered on different portions of an underlying moisture gradient. Direct evidence of the extent to which CI reflects differences in moisture availability has been lacking, however. Many factors that increase moisture availability (e.g., soil depth, silt content) also increase nutrient supply and decrease soil mechanical impedance; the ecological effects of the last have rarely been considered in any ecosystem. Decreased soil mechanical impedance should increase the availability of soil moisture and nutrients by reducing the root costs of retrieving both. Here we assess the relative importance of soil moisture, nutrient supply, and mechanical impedance in determining prairie composition and structure. We used leaf δ^13^C of C_3_ plants as a measure of growing-season moisture availability, cation exchange capacity (CEC) x soil depth as a measure of mineral nutrient availability, and penetrometer data as a measure of soil mechanical impedance. Community composition and structure were assessed in 17 remnant prairies in Wisconsin which vary little in annual precipitation. Ordination and regression analyses showed that δ^13^C increased with CI toward “drier” sites, and decreased with soil depth and % silt content. Variation in δ^13^C among remnants was 2.0‰, comparable to that along continental gradients from ca. 500–1500 mm annual rainfall. As predicted, LAI and average leaf height increased significantly toward “wetter” sites. CI accounted for 54% of compositional variance but δ^13^C accounted for only 6.2%, despite the strong relationships of δ^13^C to CI and CI to composition. Compositional variation reflects soil fertility and mechanical impedance *more* than moisture availability. This study is the first to quantify the effects of soil mechanical impedance on community ecology.

## Introduction

Curtis [1], in his seminal work *The Vegetation of Wisconsin*, established a compositional gradient for prairies of the US Midwest by calculating a *continuum index* (CI: range 100 wet – 500 dry) based on the proportions of species present from five groups of indicator taxa assumed to be associated with different topographic positions or soil types [2–4]. Curtis [1] inferred that CI reflects site position relative to soil moisture availability, and then used this assumption to study trends in the composition, structure, and environmental conditions of prairies. This approach played an important role in the development of modern community ecology, helping test the individualistic and superorganism theories of community composition, and shaping our understanding of prairies in the Upper Midwest.

One of Curtis’ key conclusions was that much of the variation in local prairie composition and structure is tied to soil moisture availability, a view largely echoed by subsequent studies [5–12]. Surprisingly, direct evidence on the extent to which Curtis’ CI reflects differences among prairies in soil moisture availability during the growing season generally remains lacking. Most studies have shown that CI is correlated with proxies of soil moisture availability (e.g., topographic position, soil texture, % organic matter, soil depth) but not soil moisture itself. At Faville Prairie in southern Wisconsin, Partch [2] did find that direct, repeated measurements of % soil moisture through the growing season were related to local differences in composition which, in turn, appeared to be tied to local topography and depth to water table as one moved from dry and dry-mesic prairies to wet sloughs. However, he did not evaluate the relationship of species composition to soil moisture vis-à-vis other environmental factors. A re-examination of Curtis’ Wisconsin data by Umbanhowar [8] demonstrated that axis 1 of an ordination of sites by species composition was closely correlated with CI, soil bulk density, water holding capacity, soil chemistry, and geographic position; axis 2 was tied mainly to differences between dry prairies on sand vs. thin soils over dolomite. Corbett and Anderson [10] found that variation in prairie composition in Illinois and Wisconsin was related to topographic position, soil texture, and soil chemistry. Almost all studies to date ascribe primacy to the effects of soil moisture, and none have quantified the relative importance of soil moisture vs. other factors in determining community composition and structure when both are included in predictive statistical models.

Few direct measurements of soil moisture availability have been made in prairies, probably because of the high cost of repeatedly measuring soil moisture at a large number of points using gravimetry, gypsum blocks, neutron probes, time-domain reflectometry, or capacitance sensors. Even if such direct measurements were made, there would remain the challenge of demonstrating that they actually represent the moisture supply tapped by plant roots [13–17]. A promising alternative approach to assessing relative moisture supply as perceived by plants uses the stable isotope discrimination signature (δ^13^C) of plant tissue. Discrimination against the photosynthetic uptake of ^13^CO_2_ vs. ^12^CO_2_ in C_3_ plants should decrease—and tissue δ^13^C become less negative—as the supply of CO_2_ relative to demand decreases in the leaf mesophyll [18,19]. As a result, δ^13^C should become less negative as moisture supply declines relative to evaporative demand. That is, drier conditions should result in a drop in stomatal conductance and an increase in δ^13^C [20–23]. In addition, drier sites are often associated with greater leaf mass and N content per leaf area, and these should also result in increased δ^13^C [24]. Studies across a wide variety of sites confirm that δ^13^C averaged across sets of C_3_ species generally increases (i.e., becomes less negative) as moisture supply relative to evaporative demand drops, as a result of local topographic position [25,26], vapor saturation deficit [27], soil moisture content [28–30], and especially annual rainfall [22, 23, 31–35]. As expected, δ^13^C of grapes at harvest increased linearly as mean pre-dawn soil water potential (i.e., soil moisture availability) between fruit set and harvest decreased across a series of vinyards and years [36].

Many factors that might increase relative moisture supply in Midwestern prairies (e.g., finer soil texture, soil greater depth) can also affect mineral nutrient availability, given the increases in cation exchange capacity and elemental concentrations in moving from sand to silt and clay fractions, and the greater rooting volume on deeper soils. This raises the key question of the extent to which shifts in prairie composition and structure along supposed gradients of relative moisture supply might instead reflect variation in nutrient availability. Coarser soil texture, lower organic content, greater bulk density, and lower moisture content also tend to increase the mechanical impedance the substrate offers to root growth, which tends to decrease root penetration and favor thicker roots with greater energetic costs per unit length [37–40]. Soil mechanical impedance (SMI) might thus reduce uptake of water and nutrients, and affect prairie composition and structure, independent of correlated shifts in moisture and nutrient supplies. The impact of soil mechanical impedance on plant communities remains, however, *terra incognita*.

Finally, one key component of prairie structure—leaf height—appears to increase, on average, from “dry” to “wet” across the Midwestern prairie continuum based on CI [5,41]. Leaf height should increase with the density of herbaceous cover in prairies [42,43] and thus with soil moisture supply, nutrient content, and depth, and decline with soil mechanical impedance. Quantitative estimates of total plant coverage in Midwestern prairies are rare, and no previous study appears to have tested our hypotheses regarding the determinants of trends in total coverage and average plant height along gradients of supposed moisture supply.

Here we test the central Curtis hypothesis—that prairies in the Upper Midwest vary primarily along a gradient most affected by moisture supply—and compare the relative impacts of soil moisture supply, nutrient concentrations, depth, and mechanical impedance on community composition and structure. We use δ^13^C in C_3_ plants as an inverse measure of soil moisture availability during the growing season, the product of cation exchange capacity times soil depth as a measure of nutrient availability, and penetrometer data as a measure of soil impedence, focusing on 17 Wisconsin prairie remnants that occupy different portions of Curtis’ compositional gradient and occur in a region with little variation in rainfall or evapotranspiration. We use CI and ordination scores as measures of community composition and putative position on a dry-wet prairie continuum, and leaf area index (LAI) and leaf height as measures of community structure. We test whether CI and ordination scores largely account for variation in each other; whether leaf height increases, as predicted, with LAI; and whether LAI increases with soil moisture, fertility, and depth, and decreases with increased mechanical impedance of the substrate. We use regression analyses to predict δ^13^C, CI, and compositional ordination scores from soil depth, texture, percent organic matter, chemistry, and mechanical impedance. Finally, we use path analyses to assess the relative proportions of compositional variation along Curtis’ continuum that can be ascribed to variation in soil moisture, fertility, and mechanical impedance.

## Methods

### Short- and long-term climate

Climate in the study region is strongly continental. From 1970 to 2000, mean annual precipitation was 875 mm; mean annual temperature, 7.8°C; mean July temperature, 22.0°C; and mean July precipitation, 100 mm [43]. Modeled potential evaporation rates averaged 900 mm annually, and increased by only 25 mm from north to south across the survey area (E. J. Hopkins, WI State Climatology Office, pers. comm.). To exclude variation in precipitation as a factor influencing δ^13^C values or community composition, we obtained 2006–2007 monthly precipitation records for seven stations across the survey area [44,45] and summed data for the 19 months prior to the survey for each station. The mean ± s.d. precipitation across stations was 1413 ± 76 mm, yielding a coefficient of variation (CV) of 5.4%, a small amount of spatial variation that would translate into only ± 0.1‰ in *expected* change in δ^13^C on three continental rainfall transects [32,46,47]. We also obtained PRISM data for each site, providing modeled average precipitation data for the 1971–2000 period on an 800-m grid [48]. These data show a long-term average of 870 ± 20 mm annual precipitation across sites, with a CV of 2.3%. There is no significant correlation of PRISM precipitation values with CI, leaf height, or estimated LAI across our sites; the correlation with δ^13^C is *r* = 0.10 (*P* > 0.66).

### Site selection and sampling protocols

**Fig 1:**
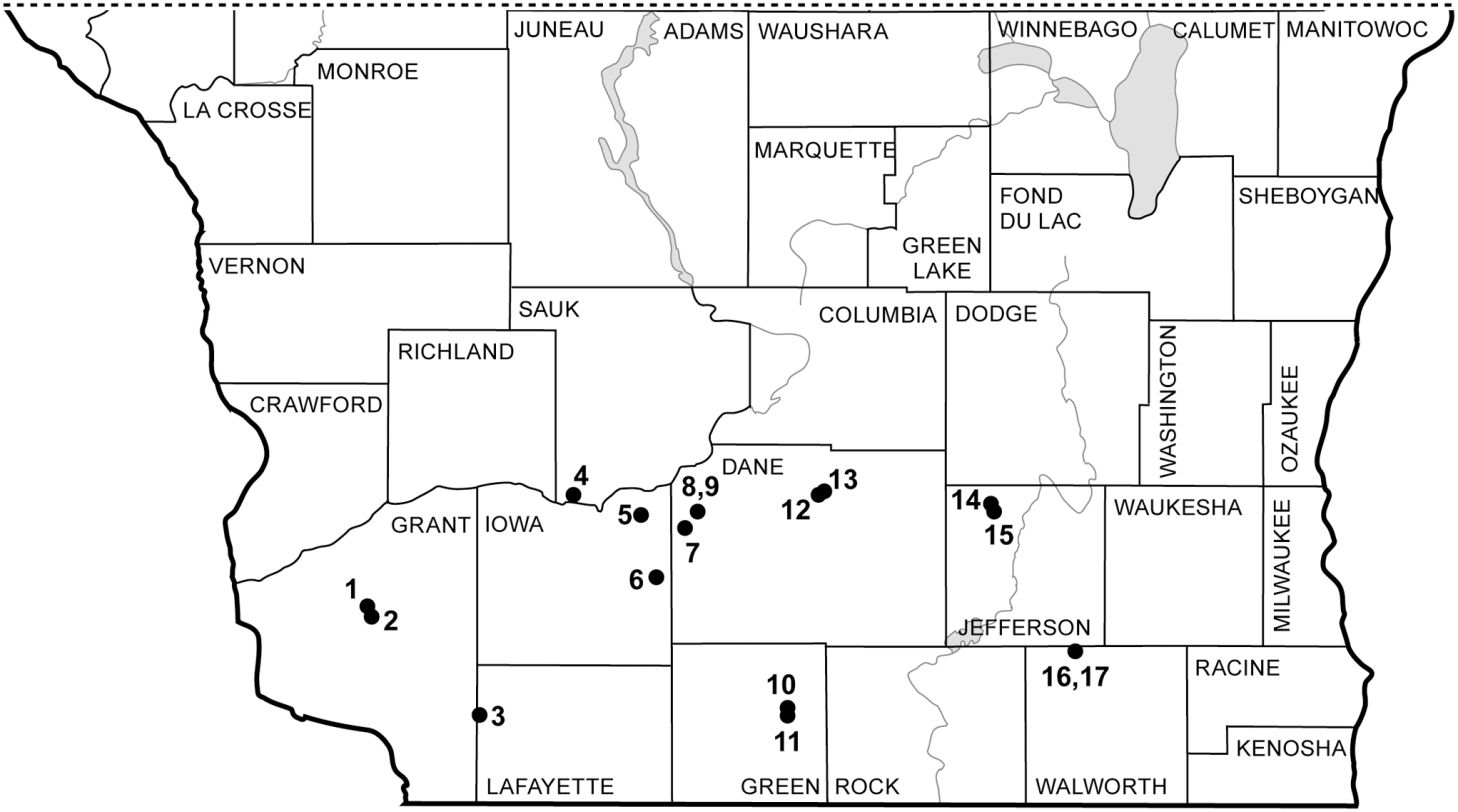
Distribution of study sites in southern Wisconsin. 1—Belcamper; 2—Bush Clover; 3—Ipswich; 4—Lone Rock; 5—Drachenburg; 6—Monroe; 7—Brock; 8, 9—Rettenmund I and II; 10—Muralt; 11—Oliver; 12—Bong; 13—Westport; 14—Snapper; 15—Faville; 16, 17—Young I and II.

Study sites were located in southern Wisconsin in a block 175 km EW by 57 km NS (43°11'N, 90°45'W to 42°41'N, 88°37’ W). We included 17 study sites (Fig 1) that each exceeded 50 m x 30 m, had a recent history of prescribed fire and brush management and no history of cultivation, exhibited little within-site variation in slope, aspect, soil type, or depth to water table, were dominated by native prairie plant species, and collectively spanned the CI continuum. Permits to study these sites were provided by Thomas Meyer of the Wisconsin Department of Natural Resources (Ipswich, Muralt Bluff, Westport, Young I and II), The Prairie Enthusiasts (Bong Road, Bush Clover, Rettenmund I and II), The Nature Conservancy (Snapper), The University of Wisconsin-Madison Arboretum (Fayville, Oliver), and four private land owners (Belscamper, Brock, Drachenburg, Monroe). One site was public land on a road right-of-way requiring no permit for study (Lone Rock). No endangered or threatened species were collected.

At each site, we established a rectangular plot 50 m long and 30–50 m wide. Plot corners were located with a Garmin 12™ GPS (accuracy ± 5 m) and flagged. Surveys of plant composition, δ^13^C, leaf height, canopy light penetration, and soil texture and chemistry were made in August to early September 2007. Plots were relocated in late July to August 2008 to repeat measurements of leaf height and identify additional species. Measurements of soil mechanical impedance (SMI) and depth were made in July to August 2010, carefully avoiding significant rainfall events.

### Species composition and CI

We tallied the presence/absence of all sterile and fertile vascular plants by species within each site, with two exceptions. Native *Carex* were identified only to genus, and a group of three visually similar, narrow-leaved panic-grasses (*Dichanthelium depauperatum*, *D*. *perlongum*, *D*. *linearifolium*) were tallied as *Panicum perlongum* to match usage in the Curtis scheme. All identifications were made by the senior author, who has years of field experience identifying prairie species, especially potentially confusing, sterile graminoids. For each site, we calculated CI following Curtis [1], producing site scores between 100 (wettest) and 500 (driest). Taxonomic nomenclature and classification of species as native/non-native followed WISFLORA (www.botany.wisc.edu/wisflora).

### Leaf height

Leaf height was estimated at 18 points scattered within each site. For grasses, leaf height was taken to be the average elevation of the uppermost leaf surface (not including flowering stems) within 0.2 m of the sample point. For forbs, leaf height was taken to be the average height of the uppermost leaves, not including any bracts on the flowering stem. Shrubs were not included, except for *Amorpha canescens*, *Rosa* spp., and *Ceanothus americanus*. The leaf height for each point within a site was the maximum of the estimates for grasses and forbs there; leaf height for each site was then calculated as the average of these point estimates. Measurements made in 2007 and 2008 were strongly correlated (*r* = 0.862, *P* < 0.0001, 2-tailed t-test with 15 d.f.) and averaged per site.

### Soil depth, texture, chemistry and mechanical impedance

Soil depth and mechanical impedance were measured *in situ* with a hand-held soil penetrometer (Penetrograph, Eijkelkamp-Giesbeek Equipment for Soil Research B.V., The Netherlands). This device continuously measures resistance pressure as a cone with a surface area of 2 cm^2^ and a relieved shaft is pushed vertically into the soil until a maximum pressure of 2.5 MPa is achieved for several seconds or the maximum length of the probe (80 cm) reached. Soil depth at each point was recorded to the nearest 0.5 cm; pressure was not recorded for soil depths < 5 cm. A total of 176 such measurements were taken over 17 sites; penetrometer soil depth was based on an average of 10.4 points per site. Special care was taken to observe regionwide weather with sampling periods conducted only during regionwide dry spells. Pressure data were transcribed at 5-cm intervals for each sample point and averaged across depths for each point, and then across points for each site to estimate soil mechanical impedance (SMI).

At each site, 12 to 18 soil cores (2 cm diameter x 20 cm deep or maximum soil depth) were collected using a bucket auger from points scattered haphazardly across the sample area. Samples at each site were then mixed, and the composite samples analyzed for % sand, silt, clay; % organic matter; cation exchange capacity (CEC, sum of milliequivalents of Ca^++^, Mg^++^, K^+^); pH; and concentrations of extractable P, K, Ca, and Mg (S1 Table). Analyses were conducted by the University of Wisconsin Soil and Plant Tissue Laboratory using methods described by [49]. On sites where deeper soils were found, samples were taken in a similar fashion from depths of 20–50 cm (7 sites), 50–80 cm (6 sites), and 80–120 cm (6 sites), pooled within the depth zone, and analyzed as above. Soil depth (≤ 120 cm) measured by auger was averaged across sample points within each site. Each soil property was averaged across the one to four depth-classes present at that site. This procedure effectively weighted the shallowest soil horizon 1.5 times as much as the second and the third shallowest horizons, and twice as much as the deepest horizon, reflecting the fact that tallgrass prairie species tend to root more densely in shallower horizons, and obtain more of their moisture (and, presumably, nutrients) from those horizons [50]. The truncation of both kinds of soil depth measurements reflects this same reality. We calculated CEC x depth as an integrated measure of cation supplies over the entire soil profile.

### Canopy transmittance and LAI

PAR (photosynthetically active radiation, 400–700 nm, in μmol m^-2^ s^-1^) was measured above the canopy and at ground level using a Decagon AccuPar ceptometer (Decagon Devices, Pullman WA) at 25 points within each site during uniformly sunny conditions. Leaf distribution parameter (Chi) was set to 1.0 with values closest to grass-dominated systems in the operator’s manual [51]. Canopy transmittance (T ≤ 1.0) for each site was calculated as the average across points of the ratio of PAR at ground level to that above the canopy. Leaf area index (LAI) was estimated assuming Beer’s Law, LAI = —ln T, and also averaged across points within sites. Modifications of Beer’s Law that assume that T scales instead as exp(-k*LAI) would simply result in estimates of LAI that differ by a factor of 1/k, and thus have only trivial effects on regressions involving LAI.

### Stable isotope analyses

The stable carbon isotope signature (δ^13^C) was measured on 94 tissue samples of C_3_ species, stratified across sites (5.5 ± 1.1 [mean ± s.d.] species per site) and species (mean = 8.0 ± 1.3 sites per species; S2 Table). We initially collected leaf tissue of ca. nine perennial herbaceous or woody species per site. Samples of woody *Cornus racemosa*, *Populus tremuloides*, and *Rhus glabra* were included if ≤ 75 cm tall and growing as isolated individuals. All plants sampled were fully exposed to sun and wind, and plants near site edges were excluded. Each species was collected by wandering through the site and taking a single leaf from each individual encountered, resulting in at least 20 leaves per species in order to maximize the spread of the sample among individuals throughout the prairie.

Samples were dried at 60°C for 4 days. From the initial 144 species-site samples, we selected 94 so that only species found in six or more prairies were included. The dried leaves in each sample were mixed, crushed manually, then remixed. An aliquot of each sample was placed in a 2-ml tube with a titanium bead; aliquots were pulverized in a Qiagen tissue lyser for 10 min, packed, and sent to the University of Arizona Environmental Isotope Laboratory in Tucson. Values of δ^13^C were measured using a continuous-flow gas-ratio Finnigan Delta PlusXL mass spectrometer coupled to a Costech elemental analyzer. Standardization was based on internal acetanilide standards calibrated against NBS-22 and USGS-24 using ratios reported in [52]. All data were expressed relative to V-PDB. Precision for δ^13^C is better than ± 0.06 ppt. Here δ^13^C = (R_sample_/R_standard_—1) x 1000 and R is the absolute ratio of ^13^C/^12^C expressed per mil or parts per thousand (‰). Mean ± s.d. values of δ^13^C were calculated for the set of C_3_ species samples submitted at each site. While species at any given site will vary in δ^13^C—perhaps reflecting differences in rooting depth, allocation to root vs. leaf tissue, specific leaf area (SLA, m^2^ leaf tissue g^-1^ dry leaf mass) [20,24]—several studies have shown that values of δ^13^C averaged across multiple C_3_ species track annual precipitation or the ratio of precipitation to pan evaporation quite well [23, 31–35, 46,47].

### Statistical analyses

Values of CI and δ^13^C were normally distributed, justifying parametric tests. Among soil variables, only [P] had to be log-transformed. We used Pearson correlations and standard linear, least-mean-squares regressions to test for significant relationships among pairs of variables (S3 Table). Simple and backward-elimination, stepwise multiple regressions were used to test for the best independent predictors of δ^13^C, CI, SMI, LAI, and leaf height, using substrate characteristics as potential predictors for δ^13^C, substrate characteristics and δ^13^C for CI and SMI, and estimated LAI for leaf height. Three separate analyses were conducted for each model for CI and δ^13^C, dropping % sand, % silt, or % clay in succession to avoid problems of collinearity, given that % sand + % silt + % clay = 1. Similarly, cation exchange capacity (CEC) was alternated in models with Ca, Mg, and K, given that CEC includes the sum of milliequivalents of those three elements. Backward-elimination models with the lowest AICc values were chosen when all variables were significant at α = 0.05; the best model among the six run for each response variable was selected based on multiple R^2^. Given the strong correlation of CI with soil mechanical impedance, and the correlation of mechanical impedance with soil depth, texture, and chemistry (see below), multiple regressions for CI were run with and without mechanical impedance as a potential predictor. Residuals by predicted plots were visually checked, found to be homoscedastic, and then checked for autocorrelation with Durbin-Watson tests. Outliers were checked with Cook’s D influence values, which for our models were all below 1.14. Residuals were tested for normality with diagnostic plots and Shapiro-Wilk W tests. All regression and correlation analyses were conducted using JMP Pro 10.0 (SAS Institute Inc., Cary, NC).

Given the strong correlation among several soil parameters, we conducted a principal components analysis (PCA) on standardized values of those parameters to identify the primary axes of covariation among them. We conducted a Bray-Curtis ordination using PC-ORD [53] to assess the primary axes of variation in vegetation composition. We applied Sørenson distance to presence/absence data and used the variance-regression technique to identify endpoints. Of the 212 species found across 17 sites, we excluded 74 that occurred at only one site, as well as the one species (*Sorghastrum nutans*) that occurred at all sites. Univariate relationships of CI, δ^13^C, leaf height, and various environmental measures to site scores on the ordination axes were evaluated in PC-ORD using joint plots, which display vectors for each variable whose components are the correlation coefficients of that variable with the scores on each ordination axis. Joint-plot vectors thus portray the direction and strength of correlations between community composition and the overlaid variables [52]. We calculated the correlations between the Sørenson distance matrices for CI, Bray-Curtis axis 1 score, δ^13^C, CEC • depth, and SMI; P values were based on Mantel tests of the null model run with 999 randomizations using PC-ORD.

Soil depth and texture are likely to affect moisture availability, cation supplies, and soil mechanical impedance. We thus used path analysis [54] to assess the relative importance of these variables in determining CI and prairie composition. We explored three models. The first involved the effects of soil depth (as measured by penetrometer) and soil texture (as quantified by % sand) on CI via soil moisture availability (as quantified by δ^13^C) and total nutrient supply (as quantified by cation exchange capacity [millequivalents of Ca^++^, Mg^++^, and K^+^ per 100 g soil] multiplied by soil depth, CEC • depth). The second path-analysis model substituted SMI for % sand as a proxy for soil texture. The third model used δ^13^C, CEC • depth, and SMI as predictors of CI. Path coefficients were calculated as standardized partial regression coefficients. The relative impact of each predictor variable on the target variable was measured using the sum of the products of the path coefficients leading from the predictors to the target.

## Results

### CI and δ^13^C in relation to each other and soil characteristics

The 17 remnants averaged 0.32 ± 0.05 ha in area, with a total of 5.47 ha surveyed. We identified a total of 212 vascular plant species, with an average of 47.1 ± 12.2 species per site. CI varied from 194.1 on the putatively wettest site to 443.8 on the putatively driest site; δ^13^C varied from -29.22‰ to -27.20‰, wi an across-sites mean of -28.08‰ ± 0.48‰ (S2 Table).

**Fig 2:**
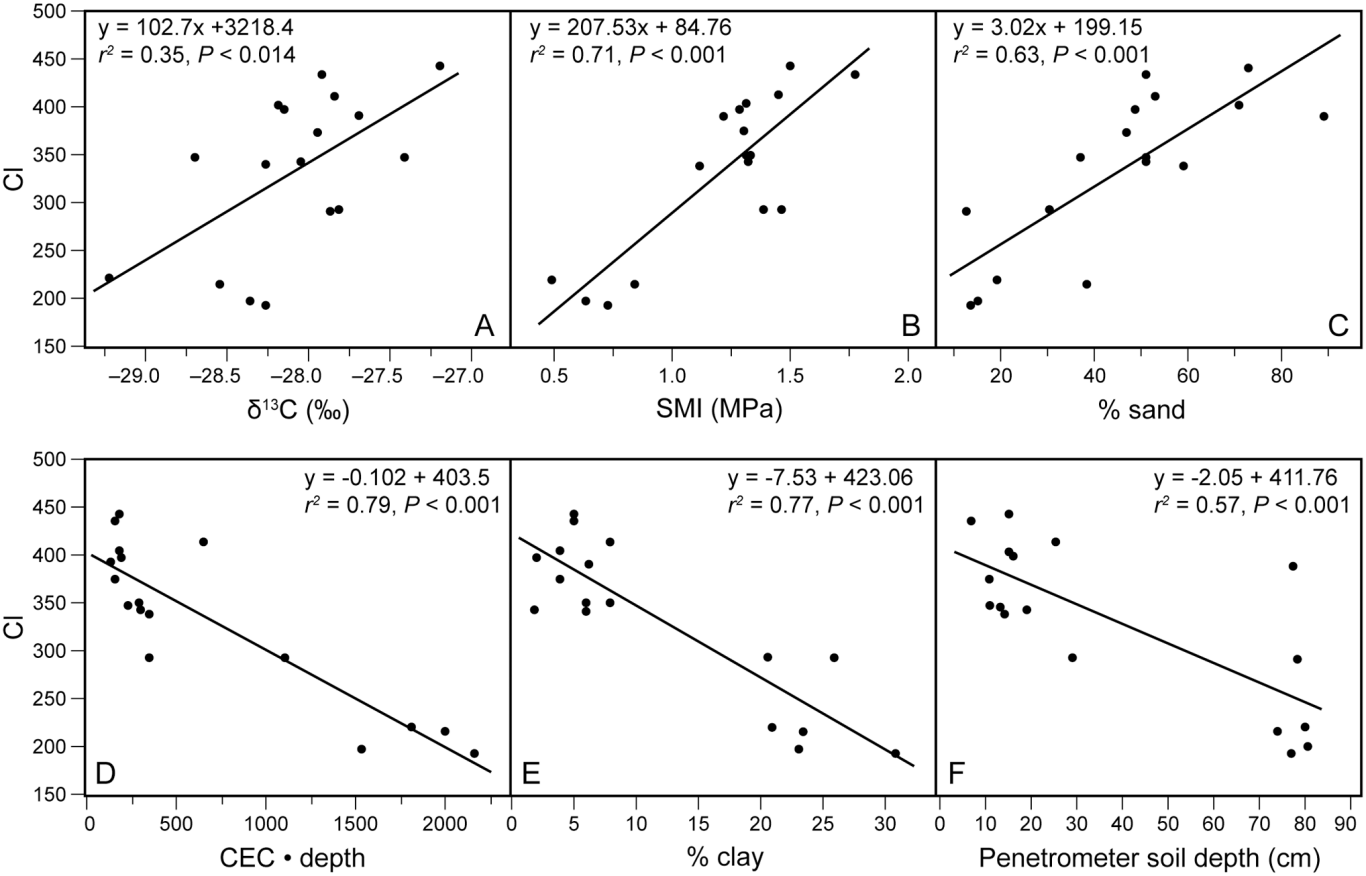
Significant increases in CI with (A) δ^13^C, (B) SMI, and (C) soil % sand; significant decreases in CI with (D) CEC • soil depth, (E) soil % clay, and (F) penetrometer soil depth. Lines represent linear regressions.

As predicted, Curtis’ CI increased significantly across sites with δ^13^C:

$$\text{CI }=\;3218.2\;+\;102.7\;•\;\delta^{13}\text{C (}‰)$$ (1)

(*r* ^*2*^ = 0.35, *P* < 0.014 for F test with 15 d.f.; Fig 2A). That is, CI—previously assumed to reflect site aridity—did, in fact, significantly increase with δ^13^C, with greater values in δ^13^C presumed to reflect lower available moisture during the growing season. Several other soil variables, however, were more closely correlated with CI than δ^13^C (*r* = 0.59). Soil mechanical impedance (SMI) and % sand both increased strongly with CI (*r* = 0.84 and 0.79) while CEC • depth, % clay, and penetrometer soil depth decreased strongly with CI (*r* = -0.89, -0.88, and -0.76, respectively) (Fig 2B–2F; S3 Table).

The best backwards-elimination multiple regression model for CI based on soil characteristics (using Ca, Mg and K, not CEC), including soil moisture via δ^13^C, indicated that CI increased with SMI and decreased with % clay and [Mg]:

CI = 274.1 + 120.0 • SMI – 4.95 • % clay – 0.04 • [Mg] (2)

(*R* ^*2*^ = 0.937, *P* < 0.0001 for F test with 13 d.f.; F ratios 30.4, 42.8, 4.8 for SMI, clay and [Mg]). That is, sites with less penetrable soil and lower clay and magnesium content are dominated more by species characteristic of drier conditions. Soil [Mg] played a small role in this model, given that SMI and % clay alone explained 91.4% of the variance in CI. When % clay is removed, a similar model appeared in which CI increases with soil mechanical impedance, % sand, and % silt:

CI = −145.1 + 118.9 • SMI + 4.26 • % sand + 3.35 • % silt (3)

(*R* ^*2*^ = 0.935, *P* < 0.0001 for F test with 13 d.f.; F ratios 28.9, 25.2, 9.0 for SMI, sand and silt). These same two models (Eqs 2–3) emerged whether or not δ^13^C is included; Eq 3 emerged whether or not CEC is included. SMI is an integrative variable: it reflects not merely mechanical impedance, but also soil depth (*r* = -0.72), CEC • depth (*r* = -0.81), % sand (*r* = 0.50), % clay (*r* = -0.61), and seasonally available soil moisture (*r* = 0.68 for δ^13^C). When we removed SMI, the best model to emerge (with or without δ^13^C) was

CI=299.7−1.6•soil depth−0.11•[Mg]+1.23•% sand+33.1•ln [P] (4)

(*R* ^*2*^ = 0.89, *P* < 0.0001 for F test with 12 d.f.; F ratios 22.7, 6.1, 7.5, 6.3 for depth, [Mg], sand, ln [P]). That is, Curtis’ inferred site aridity decreased with soil depth and magnesium concentration, and increased with % sand and phosphorus concentration. Of the environmental variables measured, SMI was the one most strongly correlated with δ^13^C (S3 Table). The best backwards-elimination model for δ^13^C based on substrate characteristics was tied solely to SMI:

$$\delta^{13}\text{C }=\;-29.248\;+\;0.97\;•\;\text{SMI}$$ (5)

(*r* ^*2*^ = 0.47, *P* < 0.0025 for F test with 15 d.f. AICc = 19.2). That is, moisture availability appeared to be lower on soils that are harder to penetrate. The best model for SMI (± CEC) was:

$$\text{SMI }=\;11.25\;+\;0.37\;•\;\delta^{13}\text{C }+\;0.15\;•\text{ ln [P] }-\;0.0044\;•\text{ soil depth}$$ (6)

(*R* ^*2*^ = 0.80, *P* < 0.0001 for F test with 13 d.f.; F ratios 15.0, 5.5, 7.7 for δ^13^C, ln [P], depth). That is, moister, deeper, more P-rich soils are easier to penetrate.

### Community structure: variation in LAI and leaf height

**Fig 3:**
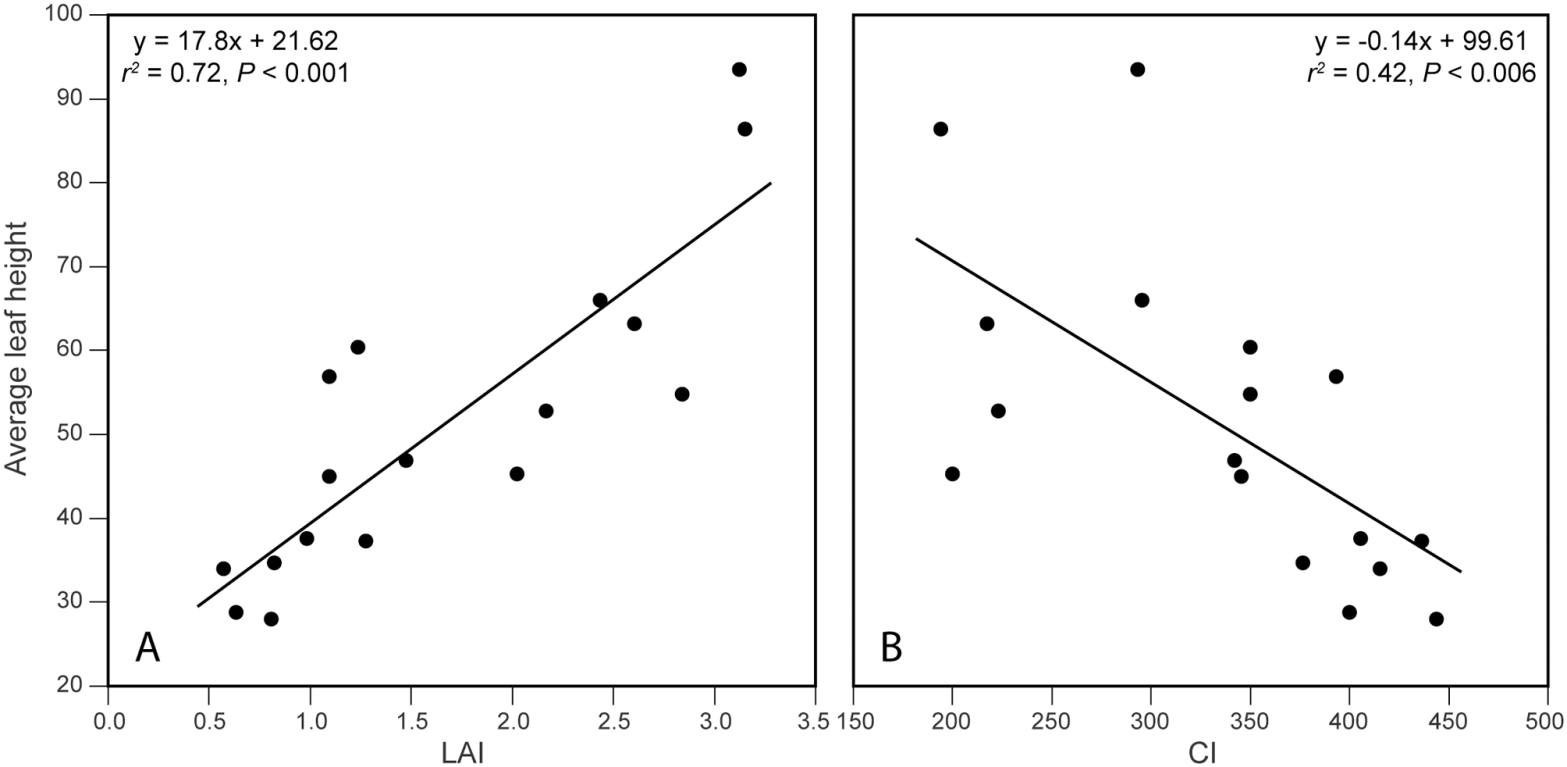
Across sites, average leaf height increased significantly with (A) LAI, and decreased significantly with (B) Curtis’ CI (100 = wettest, 500 = driest). Lines represent linear regressions.

As expected, average leaf height increased with leaf area index across sites:

leaf height (cm) = 21.62 + 17.8 LAI (7)

(Fig 3A: *r* ^*2*^ = 0.72, *P* < 0.0001, F test with 15 d.f.). Average leaf height declined significantly with increasing CI (Fig 3B; *r* ^*2*^ = 0.42, *P* = 0.005); taller plants dominated prairies considered wetter by Curtis. LAI increased with [Ca], soil depth, and % clay, and decreased with CI (*r* = 0.41, 0.59, 0.85, and -0.77, respectively). The best model for LAI in a backward-elimination multiple regression was:

LAI = 0.30 + 0.09 • % clay + 0.001 • [Mg] – 0.0002 • [Ca] (8)

(*R* ^*2*^ = 0.92, *P* < 0.0001 for F test with 13 d.f.; F ratios 113.7, 30.2, 7.4 for clay, [Mg], [Ca]). Soil moisture, as assessed by δ^13^C, did not appear in any model where all variables were significant at the 0.05% level, and was not, by itself, significant, yet simple correlation (r = -0.46) indicated that LAI increased significantly with increased soil moisture.

### Community composition in relation to soil characteristics and CI

**Fig 4:**
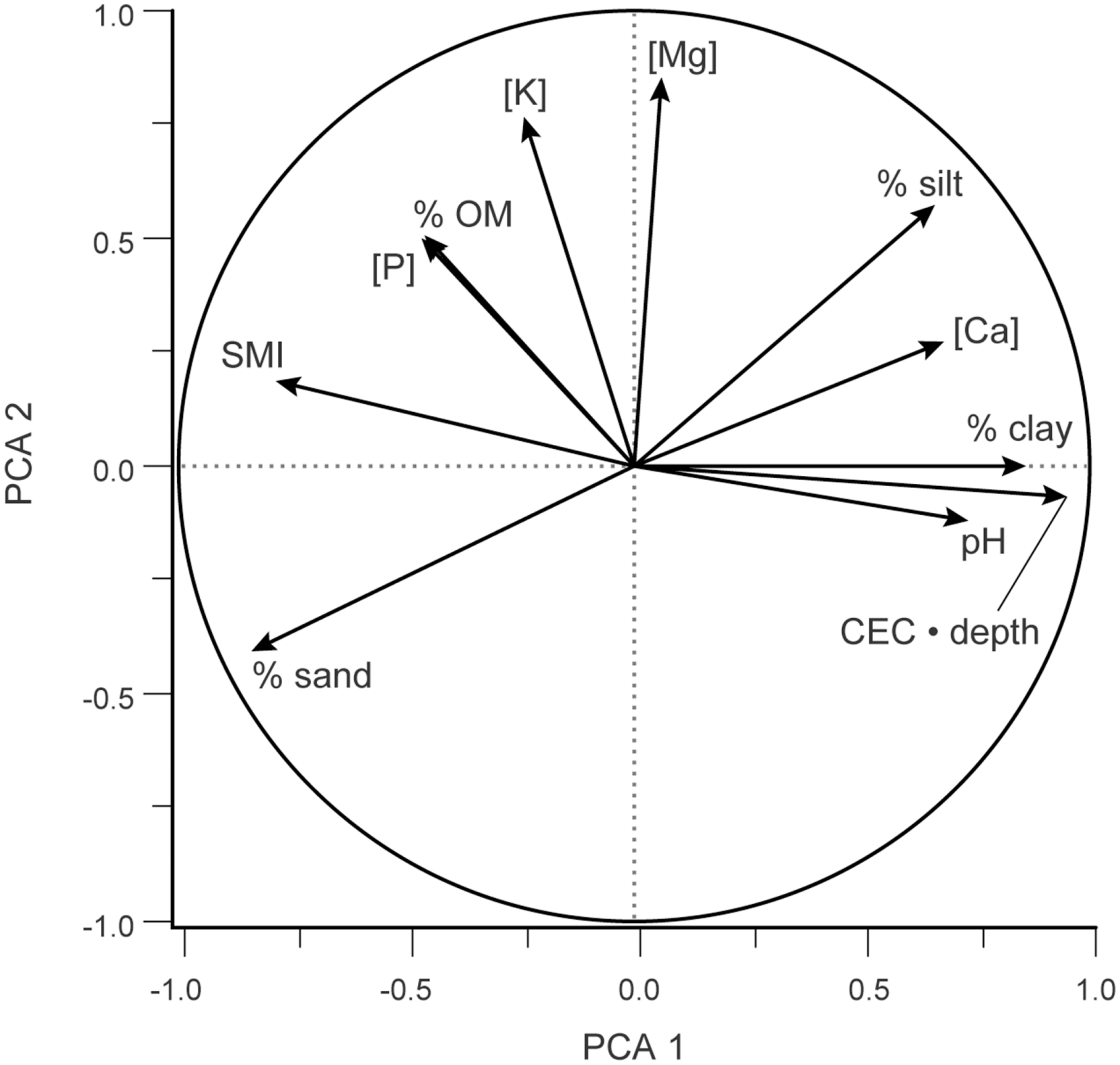
Principal components analysis on 11 soil variables; each arrow represents the eigenvector corresponding to an individual variable, surrounded by a circle of radius 1 to indicate maximum possible correlation of each variable to axes of compositional variation. Axis 1 reflects primarily CEC • soil depth and soil % clay, with soil % sand and SMI having a negative effect; axis 2 reflected mainly [Mg] and [K]. Axis accounted for 44.5% of the overall variance; axis 2, 22.2%. Total variance = 11.0 by definition.

Principal components analysis of 11 soil variables yielded three axes with eigenvalues greater than 1 (Fig 4). PCA axis 1 primarily reflected CEC • depth and % clay, and accounted for 44.5% of the overall variance in standardized soil variables; axis 2 reflected mainly [Mg] and [K], explaining 22.2% of the standardized variance. Axis 3 was associated primarily with pH, [P], and % organic matter, and accounted for 11.2% of the soils variance.

Compositional variation was coupled strongly to soil characteristics, with BC axis 1 site scores being tied tightly to those on PCA axis 1 (*r* = 0.93, *P* < 0.0001) but hardly at all to those on PCA axes 2 or 3 (*r* < 0.156, NS). Similarly, site scores on BC axis 2 were strongly tied to those on PCA axis 3 (*r* = -0.59, *P* < 0.014) but hardly at all to those on PCA axes 1 or 2 (*r* < 0.29, NS). That is, the first axis of soil variation was tightly aligned to the first axis of compositional variation, and the third axis of soil variation was tightly aligned to the second axis of compositional variation.

**Fig 5:**
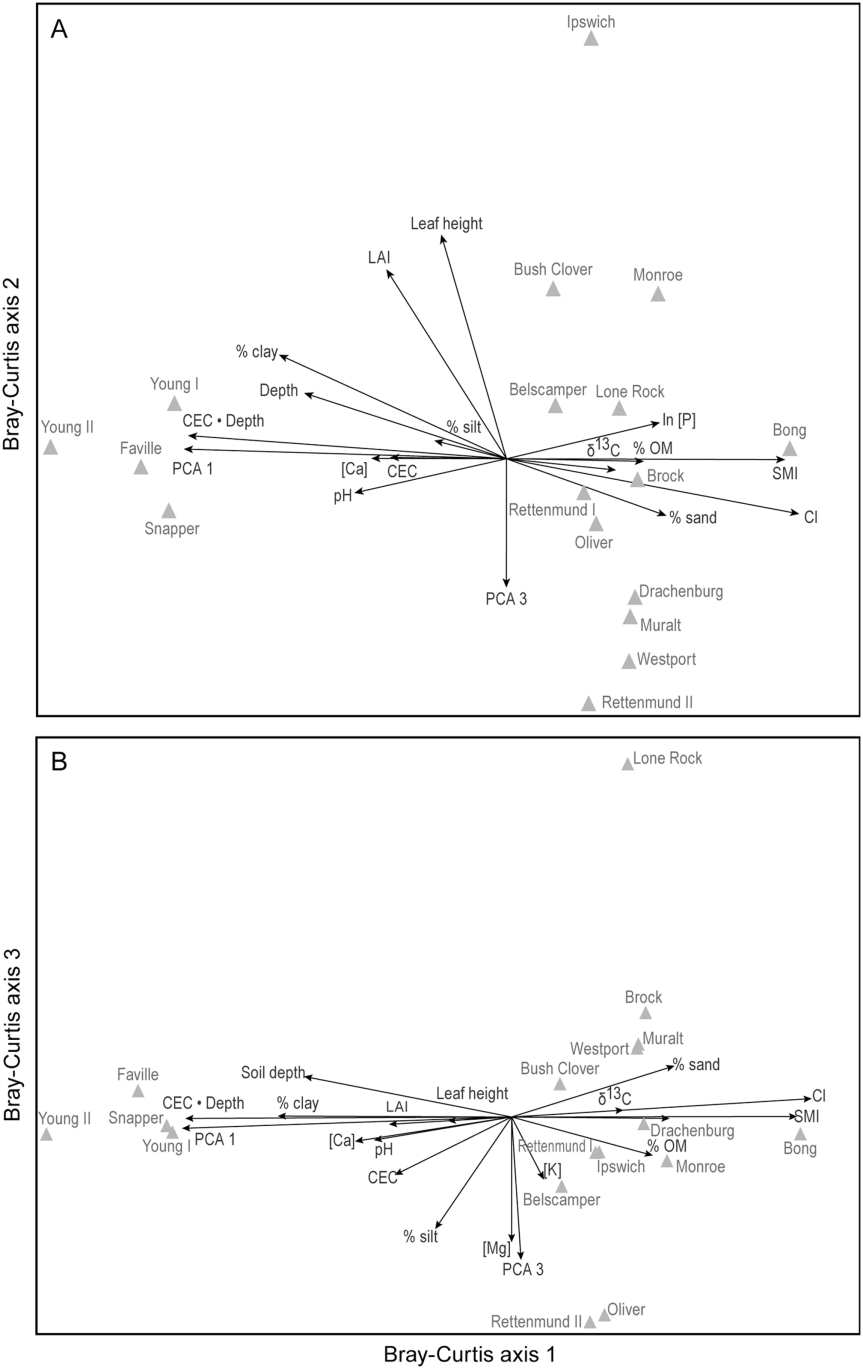
Bray-Curtis ordination of 17 sites based on presence/absence data for 137 informative species. Vectors show the strength and direction of correlations of individual environmental factors, and PCA axis 1 and 3 scores, with scores on (A) BC axes 1 and 2, and (B) and BC axes 1 and 3. Sum of squares of non-redundant distances in the original matrix = 51.084.

Bray-Curtis ordination of 17 sites x 137 informative species yielded three axes explaining 34.6%, 17.6%, and 10.2%, respectively, of the variance in compositional distance. A joint plot of CI and soil variables on the compositional ordination shows that PCA axis 1 score, CEC • depth, CI, CEC • depth, SMI, and % clay were, in order, those most strongly correlated with BC axis 1 (Fig 5A). CI itself is most strongly correlated with PCA axis 1 score, BC1, CEC • Depth, % clay, and SMI (S3 Table). BC axis 2 was most strongly positively correlated with leaf height and LAI, and negatively with PCA axis 3 (Fig 5B).

**Fig 6:**
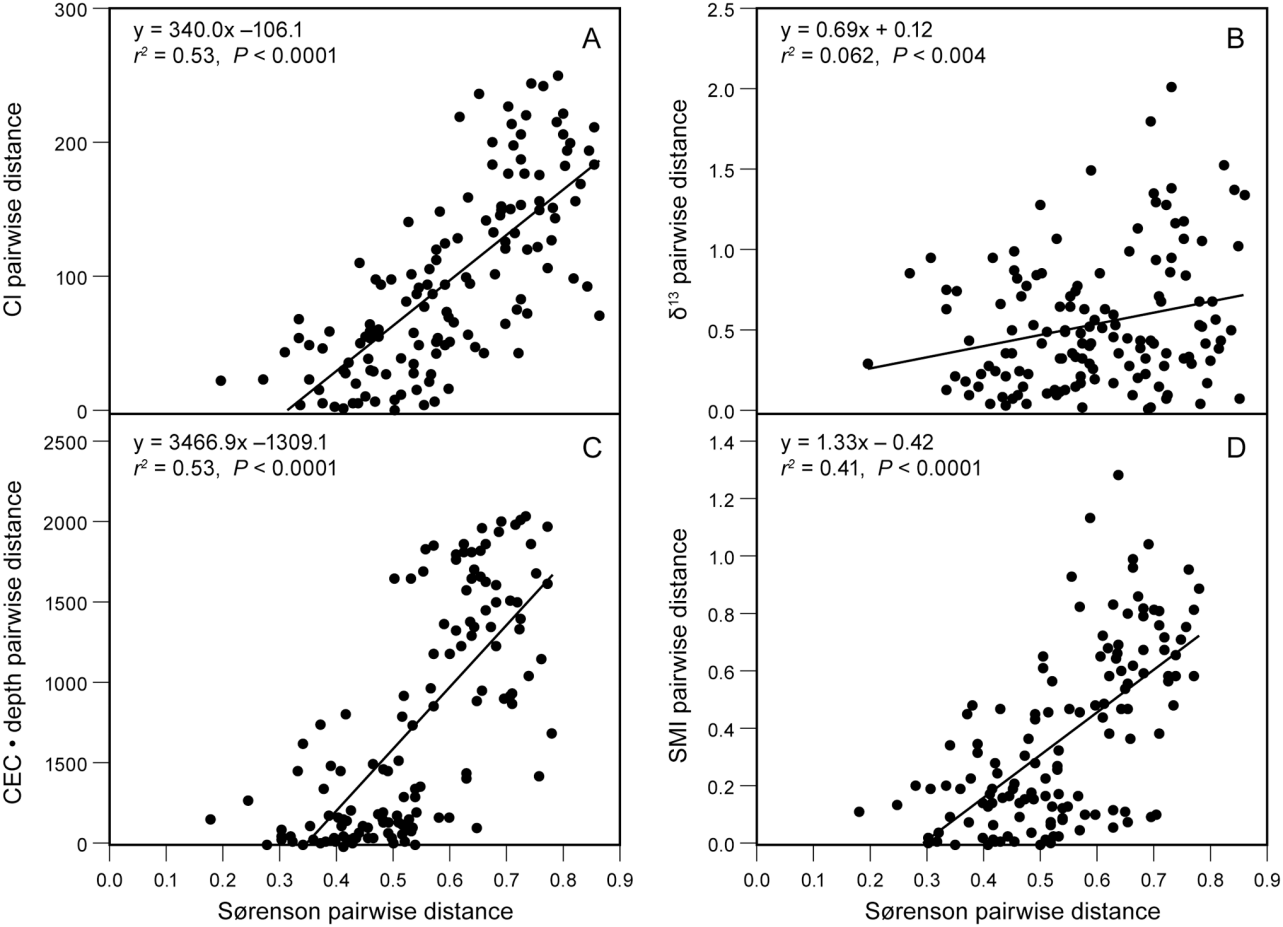
Relationships of pairwise Sørensen distances in species composition to pairwise differences in (A) CI, (B) δ^13^C, (C) CEC • soil depth, and (D) SMI. Lines represent linear regressions; significance levels are based on Mantel tests.

Variation in CI accounted for 80.0% of the variance in scores on axis 1 of the Bray-Curtis compositional ordination (S3 Table). Pairwise Sørensen distances explained only 51% of the variance in pairwise differences in BC axis 1 scores. Sørensen distances explained 53% of the variance in pairwise distances in both CI and CEC • depth, and 41% of the pairwise differences in SMI (Fig 6A, 6C and 6D; *P* < 0.001, Mantel test). Surprisingly, pairwise Sørensen distances accounted for only 6.2% of the variance in differences in δ^13^C (Fig 6B). In the pairwise relationships between CI, δ^13^C, community composition, and soil characteristics, the connection of δ^13^C to community composition was the weakest link (S3 Table).

### Path analyses: relationships among CI, moisture and nutrient supplies, and soil mechanical impedance

**Fig 7:**
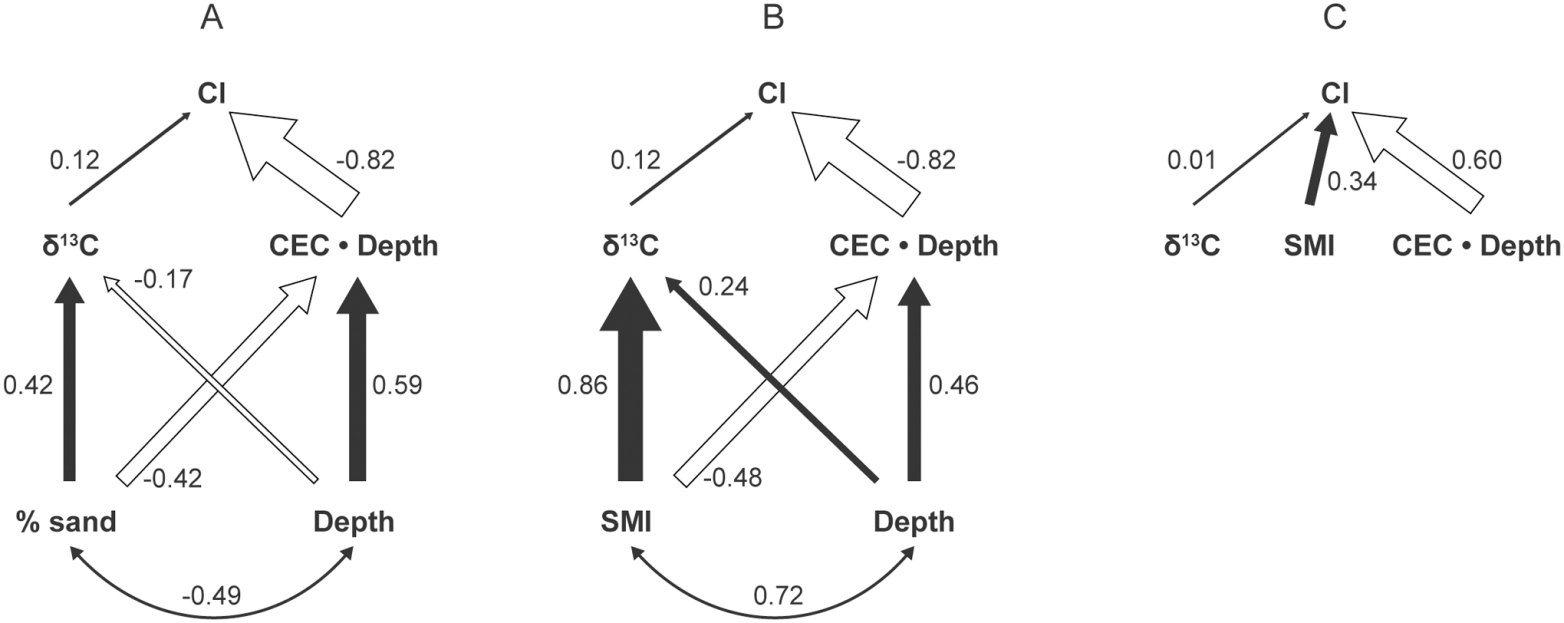
Path analyses relating effects of soil moisture, fertility, and mechanical impedance on composition as measured by CI. (A) Effects of % sand content and soil depth on CI via soil moisture supply (δ^13^C) and nutrient supply (CEC • soil depth). Numbers represent partial regression coefficients on standardized variables; the magnitude of those coefficients is proportional to arrow width, with negative effects indicated by hollow arrows. Based on the path coefficients shown, 95.9% of the effect of soil depth on CI flows through CEC • depth vs. δ^13^C; 87.2% of the effect of % sand on CI flows through CEC • depth. (B) Effects of SMI and soil depth on CI via moisture supply and nutrient supply. Based on the path coefficients shown, 79.2% of the effect of SMI on CI flows through nutrient supply; the magnitude of the effect of soil depth flowing through nutrient supply is 13.1 times that flowing through moisture supply and is opposite in sign to the former. (C) Effects of CEC • soil depth, SMI, and δ^13^C on CI.

Soil depth and texture affected the apparent supplies of both moisture and cations, with both being greater on mesic to wet prairies. However, measured variation in cation nutrient supplies appeared to have a much stronger impact on prairie composition than did that for moisture supply. Path analysis 1 indicated that % sand had equal effects, although in opposite directions, on δ ^13^C and CEC • depth (Fig 7A), yet only 12.8% of its effect on CI went through δ^13^C while 87.2% flowed through CEC • depth. An increase in soil depth increased the availability of both moisture and cations. However, only 4.1% of the effect of soil depth on CI flowed via δ^13^C, compared with 95.9% flowing through CEC • depth. With only δ^13^C and CEC • depth as predictors of CI in a multiple regression, CEC • depth accounted for 79% of the variance in CI, while δ^13^C and CEC • depth together account for only 81%; the effect of δ^13^C was not significant (*P* > 0.3).

Path analysis 2 was similar in many respects. Even though SMI had a much greater effect on moisture supply—almost double its own effect on CEC • depth and the effect of % sand of δ^13^C (Fig 7B)—the effect of SMI on CI via soil nutrients was still 3.8 times greater than its effect via soil moisture. The magnitude of the effect of soil depth on CI via soil nutrients was 13.1 times that via soil moisture (Fig 7B).

When proxies for soil nutrient supplies, mechanical impedance, and moisture content were entered in a three-way path analysis leading directly to CI, the partial regression coefficients for CEC • depth, SMI, and δ^13^C were -0.60, 0.34, and 0.01, respectively, indicating that soil moisture had by far the weakest apparent effect on CI, cation supplies the strongest, with the effect of mechanical impedance being slightly more than half that of cation supplies (Fig 7C).

## Discussion

It is natural to assume that compositional and structural variation from “wet” or “mesic” to “dry” prairies in the Midwestern North America would parallel, in many ways, the continental gradient from tall- to shortgrass prairies, which is itself clearly correlated with regional variation in annual rainfall and potential evapotranspiration. Our δ^13^C data show, for the first time, that soil moisture availability during the growing season was indeed correlated with Curtis’ CI and the local compositional continuum from “wet” to “dry” prairies in the Upper Midwest (Figs 2A and 5). Given that climate and altitude were essentially constant in south-central Wisconsin, the local 2‰ drop in δ^13^C —from -27.2‰ to -29.2‰—in moving from “dry” to “wet-mesic” prairies was dramatic and equal in magnitude to that seen in forests and acacia scrub while moving from 450 to 1600 mm of annual rainfall in north-central Australia [32], from 300 to 1200 mm rainfall in forests in southwestern Australia [46], and from 500 to 1500 mm rainfall in China [47]. On sites within a few km of each in semi-arid Utah, Dawson & Ehleringer [55] found a drop of 2.14‰ in δ^13^C leaf tissue of male *Acer negundo* trees in moving from dry uplands to mesic streamsides. In Israel, Hartman and Danin [56] found a 1.0‰ drop in δ^13^C leaf tissue of plants on five paired sites from dry exposed ridges to valley-bottom washes with greater soil moisture. These comparisons make it clear that the Wisconsin prairie continuum embodies a strong gradient in moisture availability.

But given that soil texture, depth, and mechanical impedance can affect the supplies of both moisture and nutrients, the question remains: Does soil moisture play a greater role than nutrient supply or mechanical impedance in determining plant community composition and structure in prairies? Our data provide the first evidence that, in the Upper Midwest, soil nutrients and mechanical impedance may be more important determinants of prairie composition than moisture supply, and that Curtis’ moisture continuum might thus be better seen as a fertility/impedance gradient (Figs 5–7).

In areas with subtle topography, little spatial variation in climate, and a deep water table, soil depth and texture are likely to be the primary determinants of both local moisture availability and nutrient supply. CI is closely correlated with the supply of nutrients—as measured by CEC • soil depth (*r* ^*2*^ = 0.79)—and with soil impedance—as measured by SMI (*r* ^*2*^ = 0.71), but moisture availability as measured by δ^13^C explains less than half the variance in CI (*r* ^*2*^ = 0.35). Based on path analyses, the effects of soil depth and texture on composition were 6.8 to 24 times greater via nutrient supply than via moisture supply, whereas the effects of soil depth and mechanical impedance were 3.4 to 6.8 times greater via nutrient supply than via moisture supply (Fig 7).

Because variation in soil depth and texture will, in the absence of a high water table, almost inevitably affect the supplies of both nutrients and moisture, it will often be difficult to separate the effects of moisture vs. nutrient supply. Experimental manipulation of rainfall (via irrigation or rainout shelters) has clearly demonstrated that the amount and timing of moisture supply can affect the physiology of prairie species and the net productivity and soil carbon flux of prairie communities [57–60]. The current study is, to our knowledge, the first to use comparative data to separate the effects of soil moisture vs. nutrient supply and mechanical impedance on regional variation in plant community composition.

In this study, the first two axes of compositional variation under Bray-Curtis ordination corresponded almost exactly to the first and third axes of the principal component analysis of soil characteristics, most of which are chemical or textural in nature (Fig 5). The study of Floridian grasslands by Carr *et al*. [61] appears to have been the first to use comparative data to separate the effects of climate vs. soil texture and nutrients on regional variation in plant community composition. In accord with our results, Carr *et al*. [61] found that edaphic factors were the strongest correlate of community composition, explaining 48% of the variance in composition, vs. 9% each for climate and spatial location. Similarly, Peet & Christensen [62] found that soil cation exchange capacity was a leading correlate of forest diversity in the North Carolina Piedmont. However, neither that paper nor Carr *et al*. attempted to separate the impacts of soil texture on moisture vs. nutrient availability, and neither addressed the effects of soil depth and mechanical impedance. The impact of soil texture on nutrient supply, coverage, and plant height should be incorporated into regional models (e.g., [63]) to predict the effect of shifts in rainfall and evapotranspiration due to global change.

The relatively low explanatory value of average δ^13^C for community composition might reflect moisture actually having a relatively modest impact on composition, or δ^13^C simply having an inherently noisy relationship to moisture supply. Wang *et al*. [47] found that annual rainfall (100—2000 mm) explained only 23% of the variance in δ^13^C across all growth forms and species surveyed in China. Even when Kohn [34] took annual precipitation, elevation, and latitude into account in a global survey, he was able to explain only 59% of the variance in δ^13^C. Among recent studies, Stewart *et al*. [31] found the strongest relationship of δ^13^C to annual rainfall in Queensland trees (*r* ^*2*^ = 0.70). In general, δ^13^C varies among species and growth forms at a given site; it showed a stronger relationship to species-intrinsic SLA (specific leaf area, m^2^ g^-1^) and leaf N content than to rainfall of the past season in *Eucalyptus* species from southwest Australia [24]. SLA should be greater, and leaf N content per unit area lower, in species dominating areas with higher long-term rainfall, but the connection of δ^13^C to rainfall that results may be rather weak [24].

Leaf height and coverage—two key components of prairie structure—were known in qualitative terms to increase from “dry” to “wet” prairies across the Wisconsin continuum based on CI [1,5]. We provide the first quantitative data on how both traits increase toward moister sites (i.e., those with lower CI values). Leaf height should increase with herbaceous cover in prairies [42,43], and cover should increase with increasing soil moisture, nutrient availability, and penetrability. Increases in herbaceous cover toward effectively moister sites (i.e., those with lower CI) should, in part, reflect the lower fractional allocation to roots on moister, more fertile sites [64], and—as we propose here—occupancy of more penetrable soils. Thus, leaf height, leaf area index (LAI, m^2^ leaf m^-2^ ground), soil moisture, and soil cation supply should all increase with decreasing values of CI, while soil mechanical impedance should increase with CI, as observed (Figs 2, 3 and 4; S3 Table). Our data show that average leaf height is very strongly coupled to LAI (Fig 3), as predicted by Givnish [42,43]. Although most species show some plasticity in leaf height, persistent differences in height among species may be one of the most important factors helping shape the differential distribution of species—and thus, shifts in prairie composition—along Curtis’ prairie continuum. As LAI and average leaf height increase with decreasing values of CI, in moving from infertile/dry/high impedance soils to fertile/moist/low impedance soils (Fig 3), short-statured species—such as *Anemone patens*, *Aster sericeus*, *Bouteloua curtipendula*, *Solidago nemoralis*, and other species designated by Curtis [1] as indicators of “dry” prairies—are simply not going to be able to compete for light in these productive, densely covered sites [42,64]. Conversely, tall species—such as *Aster nova-angliae*, *Spartina pectinata*, *Veronicastrum virginicum*, and other indicators of “wet” prairies—invest too much in unproductive stem tissue to have the maximum possible growth rates of species on sparsely covered sites.

This study is, surprisingly, the first to show quantitatively how closely CI correlates with prairie composition in southern Wisconsin: CI accounts for 80% of the variance in BC axis 1 scores, and 53% of the compositional distances between pairs of sites, even more than the 51% explained by BC axis scores themselves. Curtis’ CI is a remarkably successful index, integrating the effects of soil moisture, fertility, and mechanical impedance supply on prairie composition. In agreement with Umbanhowar [7], we found the greatest compositional variation uncorrelated with CI at the infertile/dry end of the continuum, involving prairies on shallow, cation-rich, relatively impenetrable soil over dolomite vs. prairies on deep sands (Fig 5A). Sites on shallow sandy soil are essentially absent in broad-scale surveys of Midwestern prairies [8,10] and sites with deeper, fine-grained soils are almost inevitably going to have moderate to abundant supplies of both moisture and nutrients under Midwestern climatic conditions.

Previous studies have shown that the composition and structure of tallgrass prairies varies with climate, herbivory, fire, soil depth, soil texture, cation and moisture supplies, N supply, water-table depth, and mycorrhizal communities [59,60,65–72]. One of our most surprising results is thus that soil mechanical impedance is a leading determinant of CI and δ^13^C. Increases in mechanical impedance should reduce root penetration thereby reducing uptake of water and nutrients from the soil, affecting prairie composition and structure independent of correlated shifts in soil moisture and nutrient supplies. Soil mechanical impedance has been ignored as a determinant of community composition and structure not only in prairies, but in almost all ecosystems. Our results suggest that its effects should be more widely investigated, and that a soil penetrometer might be added to the plant ecologist’s tool kit. Studies of soil mechanical impedance might yield new insights into the factors structuring many other kinds of communities, from tundra over permafrost to rain forests over sands vs. clays. In the immediate future, it would be interesting to study covariation in the composition and structure of grasslands with rainfall, apparent moisture availability, soil mineral nutrients, and SMI across the continental gradient from desert grasslands through shortgrass, midgrass, and tallgrass prairies in central North America.

## Supporting Information

S1 Table Soil data for 17 remnant prairies. (PDF)

S2 Table Mean ± s.d. δ^13^C and full list by species and by site, with site CI. (PDF)

S3 Table Correlations between environmental variables across sites. (PDF)
