# Supplementary material for: Relative Roles of Soil Moisture, Nutrient Supply, Depth, and Mechanical Impedance in Determining Composition and Structure of Wisconsin Prairies
Source: PLoS One. 2015 Sep 14;10(9):e0137963. doi: 10.1371/journal.pone.0137963 (PMC4569388; doi:10.1371/journal.pone.0137963)
Supplement: S1 Table — (PDF) [file pone.0137963.s001.pdf]

Table S1. Soil data for 17 remnant prairies. Organic matter (OM) and sand, silt, and clay fractions are given in percentages; penetrometer soil depth, in cm; concentrations of P, K, Ca, and Mg, in µg/g; and cation exchange capacity (CEC) = millequivalents g^{-1} of Ca^{++} + Mg^{++} + K^{+}

Name	Depth	SMI	pH	OM	[P]	[K]	[Ca]	[Mg]	CEC	Sand	Silt	Clay
Belscamper	13.9	1.1	5.0	2.0	58	78	2501	985	24	59	35	6
Bong	15.1	1.3	6.1	4.6	35	94	1283	514	11	71	25	4
Brock	25.5	1.4	6.8	3.1	13	87	4695	270	25	53	39	8
Bush Clover	29.3	1.5	6.2	3.0	15	82	1447	489	12	31	49	21
Drachenburg	10.9	1.3	6.8	3.5	24	35	1896	435	12	47	49	4
Faville	80.0	0.5	7.8	2.4	13	69	3594	589	23	19	60	21
Ipswich	78.0	1.4	6.6	2.5	58	58	2016	561	14	13	62	26
Lone Rock	76.8	1.2	5.5	1.5	23	45	193	52	2	89	5	6
Monroe	13.4	1.3	6.6	3.1	36	103	1983	1262	21	37	55	8
Muralt	6.9	1.8	6.6	3.0	41	97	2774	806	21	51	44	5
Oliver	11.0	1.3	7.2	3.1	23	111	2526	910	20	51	43	6
Rettenmund 1	19.3	1.3	7.0	2.8	13	76	2282	529	15	51	47	2
Rettenmund 2	16.6	1.3	7.1	2.8	25	72	1617	357	11	49	49	2
Snapper	80.0	0.6	8.2	1.8	9	60	2815	574	19	15	62	23
Westport	15.1	1.5	7.8	2.1	8	50	1197	298	11	73	22	5
Young 1	73.6	0.8	7.9	1.9	9	64	4041	551	27	39	38	23
Young 2	76.8	0.7	7.8	1.8	6	60	4086	596	28	14	56	31
