# Supplementary material for: Relative Roles of Soil Moisture, Nutrient Supply, Depth, and Mechanical Impedance in Determining Composition and Structure of Wisconsin Prairies
Source: PLoS One. 2015 Sep 14;10(9):e0137963. doi: 10.1371/journal.pone.0137963 (PMC4569388; doi:10.1371/journal.pone.0137963)
Supplement: S2 Table — (PDF) [file pone.0137963.s002.pdf]

Table S2. Mean ± s.d. $\delta^{13}C$ and full list by species and by site, with site CI.

Species	Mean $\delta^{13}C$	St. dev.	n
Amorpha canescens	-27.34	0.75	9
Aster azureus	-28.43	0.71	9
Aster ericoides	-28.36	0.48	9
Comandra umbellata	-27.70	0.79	6
Coreopsis palmata	-28.26	0.45	9
Cornus racemosa	-27.29	0.32	6
Euphorbia corollata	-27.29	0.72	8
Monarda fistulosa	-28.73	0.33	7
Populus tremuloides	-27.76	1.32	6
Rhus glabra	-27.30	0.50	9
Silphium laciniatum	-28.12	0.68	9
Solidago rigida	-29.60	0.59	9

Sites	Mean $\delta^{13}C$	St. dev.	n	CI
Fayville	-29.22	0.78	5	223
Monroe	-28.69	0.37	5	350
Young 1	-28.55	0.93	6	217
Snapper	-28.36	0.97	6	200
Belscamper	-28.27	0.68	6	342
Young 2	-28.26	0.84	4	194
Bong	-28.19	0.85	5	406
Rettenmund 1	-28.16	0.84	6	400
Rettenmund 2	-28.04	1.44	6	345
Drach	-27.95	0.67	6	376
Muralt	-27.92	1.17	5	436
Ipswich	-27.87	1.20	8	293
Brock	-27.84	0.50	3	415
Bush Clover	-27.83	0.85	6	295
Lone Rock	-27.69	0.79	6	393
Oliver	-27.41	0.73	5	350
Westport Drumlin	-27.20	0.38	6	444

Species	Site	Date Collected	δ13C value	Site CI
Amorpha canescens	Bong	20-Aug-07	-27.45	400.0
Amorpha canescens	Bush Clover	9 Sep 2007	-27.47	305.0
Amorpha canescens	Drachenberg	24-Aug-07	-27.90	387.5
Amorpha canescens	Ipswitch	5-Sep-07	-28.60	293.3
Amorpha canescens	Lone Rock	1-Sep-07	-27.08	392.9
Amorpha canescens	Oliver	17-Aug-07	-26.36	356.3
Amorpha canescens	Rettenmund I	22-Aug-07	-26.24	355.0
Amorpha canescens	Rettenmund II	23-Aug-07	-27.90	444.4
Amorpha canescens	Westport	2-Sep-07	-27.09	435.3
Aster azureus	Belscamper	9-Sep-07	-28.40	347.4
Aster azureus	Bong	20-Aug-07	-27.61	400.0
Aster azureus	Brock's	21-Aug-07	-28.41	418.2
Aster azureus	Fayville	30-Aug-07	-29.52	233.3
Aster azureus	Monroe	26-Sep-07	-28.58	353.8
Aster azureus	Muralt	17-Aug-07	-29.13	455.6
Aster azureus	Snapper	23-Sep-07	-28.46	200.0
Aster azureus	Westport	2-Sep-07	-27.15	435.3
Aster azureus	Young I	26-Aug-07	-28.58	205.6
Aster ericoides	Belscamper	9-Sep-07	-28.20	347.4
Aster ericoides	Ipswitch	5-Sep-07	-29.30	293.3
Aster ericoides	Lone Rock	1-Sep-07	-28.45	392.9
Aster ericoides	Monroe	26-Sep-07	-28.34	353.8
Aster ericoides	Rettenmund I	22-Aug-07	-28.34	355.0
Aster ericoides	Rettenmund II	23-Aug-07	-28.19	444.4
Aster ericoides	Westport	2-Sep-07	-27.45	435.3
Aster ericoides	Young I	26-Aug-07	-28.66	205.6
Aster ericoides	Young II	9-Sep-07	-28.27	228.6
Comandra umbellata	Bush Clover	9 Sep 2007	-28.95	305.0
Comandra umbellata	Fayville	30-Aug-07	-28.21	233.3
Comandra umbellata	Lone Rock	1-Sep-07	-26.93	392.9
Comandra umbellata	Oliver	17-Aug-07	-27.63	356.3
Comandra umbellata	Rettenmund II	23-Aug-07	-26.87	444.4
Comandra umbellata	Snapper	23-Sep-07	-27.60	200.0
Coreopsis palmata	Belscamper	9-Sep-07	-27.89	347.4
Coreopsis palmata	Bong	20-Aug-07	-28.21	400.0
Coreopsis palmata	Bush Clover	9 Sep 2007	-27.78	305.0
Coreopsis palmata	Drachenberg	24-Aug-07	-27.72	387.5
Coreopsis palmata	Ipswitch	5-Sep-07	-29.21	293.3
Coreopsis palmata	Lone Rock	1-Sep-07	-28.50	392.9
Coreopsis palmata	Monroe	26-Sep-07	-28.39	353.8
Coreopsis palmata	Oliver	17-Aug-07	-28.36	356.3
Coreopsis palmata	Rettenmund II	23-Aug-07	-28.26	444.4
Cornus racemosa	Belscamper	9-Sep-07	-27.28	347.4
Cornus racemosa	Brock's	21-Aug-07	-27.47	418.2
Cornus racemosa	Ipswitch	5-Sep-07	-26.67	293.3
Cornus racemosa	Oliver	17-Aug-07	-27.55	356.3
Cornus racemosa	Young I	26-Aug-07	-27.26	205.6
Cornus racemosa	Young II	9-Sep-07	-27.48	228.6

Euphorbia corollata	Belscamper	9-Sep-07	-28.50	347.4
Euphorbia corollata	Drachenberg	24-Aug-07	-27.23	387.5
Euphorbia corollata	Ipswitch	5-Sep-07	-27.16	293.3
Euphorbia corollata	Lone Rock	1-Sep-07	-28.26	392.9
Euphorbia corollata	Muralt	17-Aug-07	-26.53	455.6
Euphorbia corollata	Oliver	17-Aug-07	-27.16	356.3
Euphorbia corollata	Rettenmund I	22-Aug-07	-26.77	355.0
Euphorbia corollata	Westport	2-Sep-07	-26.67	435.3
Monarda fistulosa	Bong	20-Aug-07	-28.08	400.0
Monarda fistulosa	Bush Clover	9 Sep 2007	-28.76	305.0
Monarda fistulosa	Ipswitch	5-Sep-07	-28.72	293.3
Monarda fistulosa	Monroe	26-Sep-07	-28.97	353.8
Monarda fistulosa	Muralt	17-Aug-07	-28.68	455.6
Monarda fistulosa	Rettenmund I	22-Aug-07	-28.75	355.0
Monarda fistulosa	Young I	26-Aug-07	-29.15	205.6
Populus tremuloides	Fayville	30-Aug-07	-29.27	233.3
Populus tremuloides	Ipswitch	5-Sep-07	-26.49	293.3
Populus tremuloides	Snapper	23-Sep-07	-27.68	200.0
Populus tremuloides	Young II	9-Sep-07	-29.41	228.6
Rhus glabra	Brock's	21-Aug-07	-27.64	418.2
Rhus glabra	Bush Clover	9 Sep 2007	-26.94	305.0
Rhus glabra	Drachenberg	24-Aug-07	-28.06	387.5
Rhus glabra	Ipswitch	5-Sep-07	-26.79	293.3
Rhus glabra	Lone Rock	1-Sep-07	-26.92	392.9
Rhus glabra	Muralt	17-Aug-07	-26.82	455.6
Rhus glabra	Rettenmund I	22-Aug-07	-27.90	355.0
Rhus glabra	Snapper	23-Sep-07	-27.60	200.0
Rhus glabra	Westport	2-Sep-07	-27.05	435.3
Silphium laciniatum	Bush Clover	9 Sep 2007	-27.05	305.0
Silphium laciniatum	Drachenberg	24-Aug-07	-27.60	387.5
Silphium laciniatum	Fayville	30-Aug-07	-28.80	233.3
Silphium laciniatum	Monroe	26-Sep-07	-29.17	353.8
Silphium laciniatum	Rettenmund II	23-Aug-07	-28.24	444.4
Silphium laciniatum	Snapper	23-Sep-07	-28.79	200.0
Silphium laciniatum	Westport	2-Sep-07	-27.77	435.3
Silphium laciniatum	Young I	26-Aug-07	-27.79	205.6
Silphium laciniatum	Young II	9-Sep-07	-27.88	228.6
Solidago rigida	Belscamper	9-Sep-07	-29.32	347.4
Solidago rigida	Bong	20-Aug-07	-29.59	400.0
Solidago rigida	Drachenberg	24-Aug-07	-29.18	387.5
Solidago rigida	Fayville	30-Aug-07	-30.29	233.3
Solidago rigida	Muralt	17-Aug-07	-28.42	455.6
Solidago rigida	Rettenmund I	22-Aug-07	-30.24	355.0
Solidago rigida	Rettenmund II	23-Aug-07	-29.49	444.4
Solidago rigida	Snapper	23-Sep-07	-30.05	200.0
Solidago rigida	Young I	26-Aug-07	-29.85	205.6
