# Supplementary material for: Relative Roles of Soil Moisture, Nutrient Supply, Depth, and Mechanical Impedance in Determining Composition and Structure of Wisconsin Prairies
Source: PLoS One. 2015 Sep 14;10(9):e0137963. doi: 10.1371/journal.pone.0137963 (PMC4569388; doi:10.1371/journal.pone.0137963)
Supplement: S3 Table — (PDF) [file pone.0137963.s003.pdf]

Table S3. Correlations between environmental variables across sites. BC-1 and BC-2 are site scores on axes 1 and 2 of the Bray-Curtis ordination; OM = % organic matter; CEC = cation exchange capacity, Pen depth = soil depth as measured by penetrometer.

Variable	CI	BC-1	BC-2	PCA1	PCA2	PCA3	Leaf height	LAI	Auger depth	Pen depth	SMI
$\delta^{13}C$	0.59	0.55	-0.18	-0.53	-0.30	0.13	-0.13	-0.46	-0.32	-0.38	0.68
CI		0.89	-0.38	-0.90	-0.05	0.23	-0.64	-0.77	-0.74	-0.76	0.84
BC-1			-0.03	-0.93	0.16	0.06	-0.42	-0.57	-0.74	-0.74	0.87
BC-2				0.17	0.29	-0.59	0.78	0.72	0.41	0.42	-0.06
Prin1					0.00	0.00	0.49	0.65	0.71	0.72	-0.79
Prin2						0.00	0.16	0.31	-0.43	-0.38	0.19
Prin3							-0.50	-0.44	-0.37	-0.38	0.14
Leaf height								0.85	0.62	0.62	-0.30
LAI									0.60	0.59	-0.44
Auger depth										0.99	-0.69
Pen depth											-0.72

Variable	pH	O.M.	ln [P]	[K]	[Ca]	[Mg]	CEC	CEC* depth	%Sand	%Silt	%Clay
$\delta^{13}C$	-0.20	0.06	0.04	-0.07	-0.42	-0.36	-0.47	-0.57	0.51	-0.49	-0.37
CI	-0.48	0.46	0.45	0.20	-0.48	-0.17	-0.49	-0.89	0.79	-0.58	-0.88
BC-1	-0.64	0.61	0.65	0.29	-0.61	-0.02	-0.56	-0.93	0.66	-0.45	-0.79
BC-2	-0.31	-0.08	0.32	0.05	-0.01	0.23	0.05	0.25	-0.39	0.23	0.53
Prin1	0.74	-0.46	-0.61	-0.25	0.68	0.06	0.60	0.95	-0.84	0.66	0.86
Prin2	-0.12	0.50	0.50	0.77	0.27	0.85	0.49	-0.07	-0.40	0.58	0.00
Prin3	0.51	0.45	-0.50	0.33	0.29	-0.16	0.13	-0.09	0.13	-0.01	-0.29
Leaf height	0.03	-0.34	-0.02	-0.07	0.17	0.19	0.22	0.55	-0.58	0.34	0.77
LAI	0.22	-0.33	-0.10	-0.01	0.28	0.41	0.41	0.67	-0.76	0.55	0.85
Auger depth	0.38	-0.68	-0.43	-0.53	0.22	-0.32	0.10	0.80	-0.47	0.16	0.82
Pen depth	0.35	-0.64	-0.39	-0.49	0.26	-0.29	0.12	0.80	-0.49	0.20	0.81
SMI	-0.47	0.48	0.49	0.31	-0.42	-0.01	-0.39	-0.81	0.50	-0.33	-0.61
pH		-0.21	-0.74	-0.17	0.49	-0.08	0.37	0.62	-0.52	0.47	0.44
O.M.			0.39	0.51	-0.14	0.17	-0.15	-0.49	0.16	0.05	-0.45
ln [P]				0.32	-0.40	0.39	-0.23	-0.55	0.24	-0.08	-0.41
[K]					0.18	0.63	0.35	-0.26	0.03	0.11	-0.25
[Ca]						0.20	0.91	0.69	-0.53	0.47	0.46
[Mg]							0.54	0.01	-0.31	0.44	0.02
CEC								0.61	-0.52	0.48	0.42
CEC*depth									-0.72	0.48	0.88
%Sand										-0.93	-0.80
%Silt											0.52
